# Responses to Commentaries on “Ethics as if Your Life Depended on It”

**DOI:** 10.1177/15562646261473069

**Published:** 2026-07-29

**Authors:** Joshua M. Pearce

**Affiliations:** 1 Thompson Centre for Engineering Leadership & Innovation, Western University, London, Ontario, Canada

**Keywords:** research ethics, research ethics committee/IRB review, federal policies/guidelines/office of human research protections, otherinternational clinical research ethics, research ethics, RCR

## Abstract

Responses to Dr. Robert Steel and to Dr. Min-Fu Tsan.

## Response to Dr. Robert Steel

I would like to thank Dr. Robert Steel, for the kind and thoughtful review of my article “*Ethics as if your life depended on it*” (Pearce, 2026). We can both agree that ethics delays that impose mortality costs that vastly exceed the harm they prevent are simply unacceptable. We share the frustration that the regulatory community has known the fundamentals of this problem for half a century, while human lives were (and continue to be) sacrificed to poor ethics policy. Although we agree in spirit, Steel provides five main reservations regarding my arguments for democratizing ethics oversight and altering the current medical research framework.

First, Steel questions whether democratization is appropriate for addressing the outlined problems, expressing concerns about conflicts with expert oversight and the potential burden on patients to judge evidence or make technical decisions. However, my framework does not eliminate expert roles and does not take the rails off. It simply redistributes decision-making power to patients for their own risks while preserving expert input through independent safety monitoring boards. Transparency and structured consent tools, supported by studies like Blom et al. (2025) and Nishimura et al. (2013), empower patients without overburdening them, ensuring that they can make informed choices or defer judgment to experts when necessary. By addressing the ethical asymmetry outlined in the Belmont Report, my approach strikes a balance between patient autonomy and expert guidance, enabling collaborative decision-making without creating undue hurdles.

Next, Steel distinguishes between objectionable and unobjectionable paternalism, arguing that some paternalistic measures are beneficial. My critique of paternalism is grounded in the idea that competent adults should be empowered to make decisions about their own health risks, even under extreme conditions. Gianella et al. (2017) highlight that individuals nearing the end of life often value participation in research despite risks. This suggests that paternalistic restrictions can undermine autonomy and meaningful choices for these individuals. My framework respects informed patient decisions while still providing safeguards through transparency and independent oversight.

Steel points out that there is “limited empirical evidence” for the efficacy of engagement strategies like adaptive trial designs, but there is evidence that adaptive clinical trial designs reduce timelines and improve outcomes for high-risk interventions (Mulligan, 2021).

Steel's strongest arguments concern the effects on others, such as delayed data collection and financial burdens. Accelerating trials for terminally ill patients, however, allows faster real-time data collection during post-market surveillance, balancing urgency with safety, as seen in Japan's regenerative medicine model (Act on the Safety of Regenerative Medicine, 2026). Financial burdens can be mitigated by adapting payment systems to avoid socializing the costs of experimental treatments. By offering tiered pricing models or requiring pharmaceutical companies to bear the costs during early stages, democratization can still achieve equitable access while addressing Steel's concerns.

The greatest risk, however, is perhaps those of unintended consequences. Steel highlights that recommendations such as adaptive trial designs and increased patient governance could inadvertently lead to additional bureaucratic complexity. This complexity could further delay research timelines, undermining the goal of faster drug approval. The evidence from Operation Warp Speed (Winch et al., 2021), however, demonstrated that bypassing traditional bureaucratic obstacles accelerated vaccine development without compromising safety. This shows that reforms can balance efficiency and safety. Post-market surveillance ensures safety while allowing timely access to experimental drugs. There is no reason to continue to sacrifice human lives to antiquated research ethics policies and practices.

Steel highlights potential conflicts between individual autonomy and broader societal costs, such as delays in generating reliable safety and efficacy data when off-trial access to experimental drugs is allowed. I am arguing that we alter the research ethics framework to allow access to experimental drugs as part of trials. I recommend that we use adaptive trial designs, patient governance, and transparency, but Steel worries that these could potentially create new bureaucratic hurdles, further delaying research timelines. Neither of us want more delays, of course. There is no evidence that adaptive trial design or transparency would create delays. In fact, all of the evidence from open source technology development points to substantial acceleration of timelines (Pearce, 2020; Raymond, 1998). It is also intuitively obvious that allowing patient governance would only speed up the process. To prove this, I present the following thought experiment*:*

Imagine a terrorist leader contracts a blood-borne fatal disease with no known cure. He grows angry as he watches fellow patients die as a local research ethics review board (REB) delays clinical trials of new potential cures. He meticulously investigates the REB members, draws his own blood and then with the help of his terrorist group manages to invade the homes of all REB members in the same night and inject them with his disease. If this were to occur, it should be clear to readers that the REB members would change their decision-making process and priorities substantially. I argue here that REBs impose complex bureaucratic hurdles that delay trials and research approvals. Personal stakes might drive REB members to reduce these hurdles, favoring adaptive trial designs and democratized oversight, which have been shown to accelerate timelines (Mulligan, 2021). With their own lives on the line, they would likely prioritize faster approval processes to access potentially life-saving treatments earlier. For example, REBs typically meet monthly for full-board reviews of higher-risk studies. It is highly likely that the infected REB members would choose to meet more frequently, as many did during the COVID-19 pandemic (see Salamanca-Buentello et al., 2024). In addition, personal experience with the disease might shift REB members’ perspectives to accept higher levels of risk in exchange for potential benefits, aligning with my argument that terminal patients often prioritize access over safety (Brett Hauber et al., 2013; Janke et al., 2024). Finally, facing the disease could increase empathy for patients, leading the members of the REB to place greater weight on patient autonomy and preferences.

This thought experiment clarifies that patients with fatal diseases (including if they are REB members) are more likely to choose the new model if given the choice because it aligns with their urgency for treatment, desire for autonomy, and willingness to accept risks. Peer-reviewed studies support this perspective (Assouline et al., 2021; Brett Hauber et al., 2013; Janke et al., 2024), especially in contexts like terminal illnesses and pandemics, as we all just experienced with COVID-19, where delays are life-threatening. It is time we started treating ethics as if our lives depended on it.

## Response to Dr. Min-Fu Tsan

In the response to my article “*Ethics as if your life depended on it*” (Pearce, 2026), Dr. Min-Fu Tsan makes four main claims in defense of the status quo: (1) that I provide “no data” that IRB review delays clinical trials; (2) that the 2018 revised Common Rule's single-IRB (sIRB) mandate has eliminated multi-IRB delays; (3) that my framework rests on a “therapeutic misconception”; and (4) that the Right to Try Act already provides sufficient access for terminal patients.

Before addressing these, I must clarify the scope of my argument: my claim is not that IRBs *alone* explain all regulatory delay, but that they are a documented, non-trivial contributor to trial start-up delay, enrollment restriction, and access friction within a broader regulatory system whose cumulative effects impose severe mortality costs. Below, I evaluate each of Dr. Tsan's claims, identify their empirical and logical errors, and provide peer-reviewed support for my original argument.

### The Empirical Reality of IRB Delays

Tsan's first claim (that there is “no evidence” that IRB review delays clinical trials) mischaracterizes the text and ignores the cited literature. My original commentary explicitly cites several peer-reviewed articles that clearly show that IRB delays slow clinical trials. These include: (Eichler et al., 2013; Isakov et al., 2019; Mo et al., 2025; Mulligan, 2021; Peltzman, 1973; Shiga et al., 2025; Taguchi et al., 2025; Uyl-de Groot et al., 2020; Wardell, 1974; Winch et al., 2021). Tsan conflates “no data I accept” with “no data exists.” Rather than engage with any of the substances of my article or any of the many citations, Tsan simply ignores them. There is also a clear internal contradiction to Tsan's argument that first states there is “no evidence”, then immediately concedes that “in the past, the IRB ethics approval system might have caused some delay” thereby contradicting Claim 1.

The reality is that the delays continue: A massive multi-site study found the median number of days from the kickoff call to cIRB approval was 216 days (Stephens et al., 2025). Similarly, Abbott and Grady (2011) document systematic IRB-driven delays and inter-IRB inconsistency (paradoxically also cited by Tsan). De Jonge et al. (2021) found the median time from the first submission to a regulatory authority to initiation of a stroke trial site was 784 days. Perhaps the strongest argument comes from the “Real-Time IRB” study that reduced IRB turnaround from 63 days to 18.8 days, directly proving that IRB-driven delay exists and is remediable (Spellecy et al., 2018). I could go on, but anyone that has done any kind of human subjects research knows that one thing IRBs do not do is speed up research. The empirical evidence that the current IRB system slows research is overwhelming, and these delays result in human deaths, yet the status quo remains after decades and unnecessary human deaths continue.

### The Persistent Failure of the sIRB Mandate

Tsan's second claim (that the 2018 revised Common Rule solves the problem) fails on four levels: 1) It conflates rule promulgation with rule effectiveness (e.g., a rule mandating sIRB existence does not establish that delays have been eliminated), 2) It ignores a plethora of published evidence to the contrary as multiple post-2018 studies show persistent and in some cases unfortunately even increased burden after sIRB implementation (Corneli et al., 2021; Green et al., 2023; Lidz et al., 2018), 3) Tsan has a domain restriction error: sIRB applies only to U.S. federally funded multi-site studies; it does not cover FDA-regulated industry trials (the majority of clinical research), single-site studies, or international research, 4) There is an obvious non-sequitur regarding the drug lag because even if sIRB eliminated all IRB delays inside the U.S. (which it most certainly has not) it would not address EMA in Europe, PMDA in Japan, NMPA in China, etc. drug lag (see the Mo, Uyl-de Groot, Shiga, Taguchi articles I originally cited).

For an example of the literature in this regard, Murray et al. investigated the IRB burden in 2021 (Murray et al., 2021). Later investigation on whether the sIRB solved the problem concluded “many of the anticipated benefits of the policy have yet to be realized” and that sIRB has often “shifted or even increased burden rather than reduced it” (Green et al., 2023, p. 5). Green et al. document duplicative local reviews, lack of harmonization, and start-up delays. That was in 2023, which is five years after the sIRB was implemented. Lidz et al., similarly found more empirical evidence that sIRB reliance arrangements create new bottlenecks (Lidz et al., 2018). Corneli et al. also found delays with the sIRB (Corneli et al., 2021). In summary, the empirical record post-2018 directly contradicts Tsan's claim that sIRB has “solved” IRB-driven delays.

### Therapeutic Misconception and Institutional Control

Tsan's third claim is a misapplication of the therapeutic misconception which describes patients’ mistaken beliefs (false hope) that research is individualized treatment (Appelbaum et al., 1987). It is a well-known cognitive error attributed to *participants*, not a category mistake committed by a policy author who is not a participant (me). In my piece, I explicitly frame trials as research with uncertain outcomes; I do not assert that trials are treatment. Invoking the doctrine against an autonomy-based policy argument is a categorical misuse. Tsan's response uses “therapeutic misconception” as a rhetorical shield to deny that *some* trial participation does, in fact, confer therapeutic benefit (especially in oncology accelerated-approval and expanded-access pathways (Fountzilas et al., 2018; Wong et al., 2024)). For terminally ill patients with no alternative, the distinction between “research” and “last-resort therapy” is, as a matter of lived experience, blurred. The doctrine itself has been contested on precisely these grounds and Hordijk et al. (2022) directly challenge paternalistic deployment of “false hope”. Melo-Martín and Ho show the doctrine is overextended when used to override autonomy (2008). Perhaps most importantly, this is paternalism dressed as ethics. Treating the patient's framing of trial participation as a “misconception” to be corrected by an IRB is exactly the autonomy infringement I am critiquing. Tsan's response thus illustrates rather than refutes my argument. Finally, this view is inconsistent with the Right to Try Act itself, which Tsan invokes (Claim 4 below): if trial-as-therapy is always a misconception, the Right to Try Act would itself be incoherent.

One element of Tsan's third claim warrants direct reply: the observation that sponsors, not IRBs, write protocols, and that in randomized controlled trials, participants may be assigned to the control arm. Both points are correct and neither rebuts my framework. My proposed framework does not ask IRBs to author eligibility criteria; it asks them to reform the gatekeeping functions they already control (consent thresholds, adaptive-design approval, transparency requirements, and Data Safety Monitoring Board (DSMB) composition). The empirical literature shows that IRBs in fact materially shape eligibility through required protocol modifications (Abbott & Grady, 2011; Silberman & Kahn, 2011), so the clean sponsor-versus-IRB dichotomy that Tsan relies on does not hold. As for randomization: my commentary does not claim that trial enrollment guarantees the active agent. It claims that for a terminally ill patient, a non-zero probability of access under monitored conditions is preferable to the zero probability outside the trial, and that the autonomous adult is the right person to weigh that probability. Tsan's structural objection (that meaningful access depends on sponsor behavior as well as IRB behavior) is in fact an argument *for* my proposed five-part framework rather than against it, because the framework targets sponsor incentives (open-source transparency, post-market surveillance, patient-representative DSMBs) as well as the IRB process.

### The False Sufficiency of Right to Try

Finally, Tsan claims that the Right to Try Act resolves the issue. This is false sufficiency. The Right to Try Act has been shown to be largely inoperative because it addresses neither manufacturers’ willingness to supply, nor insurance coverage of, the drugs in question. The Right to Try Act resulted in only a handful of patients per year accessing drugs via the Act (per FDA annual reports to Congress), in contrast to thousands per year via Expanded Access. I already expressly addressed and refuted this when I wrote that the Right to Try “is far too narrow in scope, applies only to drugs already in the pipeline, and does nothing to address the broader structural problem” (Pearce, 2026). Tsan's comments do not engage this counter-argument, but simply re-assert that my claim is already rebutted. Worse still, it seems myopic because it ignores international drug lag as the Right to Try is U.S.-only and I explicitly cited mortality estimates outside of the U.S. (e.g., (Mo et al. (2025) for the China lag, Shiga et al. (2025) for the Japan lag and Uyl-de Groot et al. (2020) for EU losses).

Even considering just the U.S. case, as I originally pointed out, Isakov et al. (2019) formally demonstrate that current significance thresholds are far more conservative than welfare-optimal for terminal illness, which Right to Try does not remedy. Bateman-House and Robertson (2018) also argue that the Act fails to expand meaningful access and may worsen patient protections. Mahant (2020) shows that it has resulted in negligible patient access. Finally, Mulligan (2021) proves that the mortality cost of regulatory caution dwarfs the narrow Right-to-Try carve-out.

Finally, Tsan fails to address Operation Warp Speed or distributed open-source manufacturing, which are central pillars of my argument. We have just witnessed that when decision-makers perceived their own lives to be at stake, we move research much faster than the status quo and saved human lives. Tsan ignores the Belmont autonomy argument. My normative core is that respect-for-persons provides an ethical basis for patient-led decision-making for the terminally ill; Tsan's response substitutes a procedural defense of IRB authority for the moral argument. Finally, his response concludes that my framework would reduce human subject protections and research quality but provides no mechanism nor evidence of this. This is a non-sequitur given Tsan's own admission that my framework retains DSMB, post-market surveillance, and independent monitoring.

## Conclusions

Steel's commentary raises valid concerns, but my model ultimately provides a more equitable and effective solution for addressing the ethical failures of the current system. By prioritizing autonomy, transparency, and urgency, my framework ensures that the voices of those most affected by fatal diseases are heard and respected. The evidence that I cite supporting adaptive trial designs, post-market surveillance, and enhanced consent processes demonstrates that the risks raised in Steel's commentary can be mitigated. Overall, the proposed new framework could reduce drug approval timelines by years, significantly accelerating access for patients with fatal diseases. While risks exist, evidence from COVID-19 trials and adaptive designs suggests that these can be managed effectively through transparency and post-market surveillance. For patients facing life-threatening conditions, the democratized ethics model offers hope and justice that the standard model often fails to deliver (Pearce, 2026).

On the other hand, Tsan's defense of the status quo fails at three levels. Empirically, it denies IRB delays that the literature shows plainly exist. Conceptually, it misapplies therapeutic misconception to a normative argument about institutional authority. Ethically, it does not answer the central question: why should terminally ill, informed consenting adults be denied greater authority over risk when the cost of caution is often death? My commentary, while perhaps normatively bold, is empirically defensible. Tsan's response, in contrast, contains internal contradictions, ignores cited evidence, and substitutes assertion for engagement. As I clearly showed in the original commentary, IRB delays result in more human mortality than they protect (Isakov et al., 2019; Mulligan, 2021). The current system is not logically defensible, and it is long past time that we fixed it.
